# Normal distal pulmonary vein anatomy

**DOI:** 10.7717/peerj.1579

**Published:** 2016-01-14

**Authors:** Wiesława Klimek-Piotrowska, Mateusz K. Hołda, Katarzyna Piątek, Mateusz Koziej, Jakub Hołda

**Affiliations:** Department of Anatomy, Jagiellonian University Medical College, Krakow, Poland

**Keywords:** Myocardial sleeves, Middle lobe vein, Atrial fibrillation, Morphometry, Heart anatomy

## Abstract

**Background.** It is well known that the pulmonary veins (PVs), especially their myocardial sleeves play a critical role in the initiation and maintenance of atrial fibrillation. Understanding the PV anatomy is crucial for the safety and efficacy of all procedures performed on PVs. The aim of this study was to present normal distal PV anatomy and to create a juxtaposition of all PV ostium variants.

**Methods.** A total of 130 randomly selected autopsied adult human hearts (Caucasian) were examined. The number of PVs ostia was evaluated and their diameter was measured. The ostium-to-last-tributary distance and macroscopic presence of myocardial sleeves were also evaluated.

**Results.** Five hundred forty-one PV ostia were identified. Four classical PV ostia patterns (two left and two right PVs) were observed in 70.8% of all cases. The most common variant was the classical pattern with additional middle right PV (19.2%), followed by the common ostium for the left superior and the inferior PVs (4.44%). Mean diameters of PV ostia (for the classical pattern) were: left superior = 13.8 ± 2.9 mm; left inferior = 13.3 ± 3.4 mm; right superior = 14.3 ± 2.9 mm; right inferior = 13.7 ± 3.3 mm. When present, the additional middle right PV ostium had the smallest PV ostium diameter in the heart (8.2 ± 4.1 mm). The mean ostium-to-last-tributary (closest to the atrium) distances were: left superior = 15.1 ± 4.6 mm; left inferior = 13.5 ± 4.0 mm; right superior = 11.8 ± 4.0 mm; right inferior = 11.0 ± 3.7 mm. There were no statistically significant differences between sexes in ostia diameters and ostium-to-last-tributary distances.

**Conclusion.** Only 71% of the cases have four standard pulmonary veins. The middle right pulmonary vein is present in almost 20% of patients. Presented data can provide useful information for the clinicians during interventional procedures or radiologic examinations of PVs.

## Introduction

The pulmonary veins (PVs) are large blood vessels that carry oxygenated blood from the lungs and drain into the left atrium (LA) of the heart. Most individuals have four pulmonary veins, two on the left and two on the right (the inferior and the superior one), but there are also different anatomical variations. The transition between the LA and PVs (the venoatrial junction) is smooth without any pronounced folds. Moreover, there is no microscopic boundary between the PVs and the LA endocardium (Moubarak et al., 2000). Thus, the anatomical identification of the PV ostium limits, both in the corpse and using imaging techniques in living subjects, is usually complicated.

Despite the fact that PVs are the shortest of the great vessels, we cannot deny their important physiological and pathophysiological role. Publications around the world warn that ectopic centers responsible for the initiation of atrial fibrillation (AF), which is the most common cardiac arrhythmia, could be located in PVs (Fynn & Kalman, 2004; Haïssaguerre et al., 1998; Jaïs et al., 1997). The major sources of these ectopic beats appear to be the muscular (myocardial) sleeves of the distal PVs which are simple extensions of the left atrial myocardium over the outer surface of PVs (Saito, Waki & Becker, 2000; Woźniak-Skowerska et al., 2011). This is the reason why they became a target of interventional cardiology procedures such as catheter radiofrequency pulmonary vein isolation (Chen et al., 2000; Gill, 2004). Hence, anatomy and morphology of PVs are crucial for planning and performing invasive procedures by electrocardiologists and surgeons. It leads us to the aim of this study, which was to present normal distal PV anatomy and to create a list of all PV ostium variants that can be useful for clinicians. Also the ostium-to-last-tributary distance and macroscopic presence of myocardial sleeves were evaluated.

## Methods

### Study population

This study was conducted by the Department of Anatomy, Jagiellonian University Medical College and it was approved by the Bioethical Committee of the Jagiellonian University Medical College, Cracow, Poland (KBET/51/B/2013). Hearts were collected from deceased persons when no objection was expressed - both from the donor and family. We examined PVs in 130 randomly selected autopsied human hearts (Caucasian) of both sexes (94 males, 36 females) aged from 17 to 94 years old (49.2 ± 18.2 years old) with an average measured body mass index (BMI) of 27.2 ± 6.0 kg/m^2^. All heart specimens were precisely collected during routine forensic medical autopsies performed in the Department of Forensic Medicine, Jagiellonian University Medical College from July 2013 to October 2014. Exclusion criteria include: severe anatomical defects, the overall condition of the heart after heart surgery and grafts on the heart, visible severe macroscopic pathology of the heart or vascular system found during the autopsy (aneurysms, storage diseases), heart trauma, and macroscopic signs of cadaver decomposition. The main causes of death were: suicide, traffic and home accidents, as well as murders. No death due to heart failure was observed. There were no cases with past medical history of AF in the study group.

### Dissection and measurements

All hearts were removed together with the proximal portions of the great vessels: the ascending aorta, the pulmonary trunk, the superior vena cava, the inferior vena cava, and with all PVs in particular. The preparation of the PVs was conducted from their ostia to the LA, along their course towards the hilum of the lung. The main trunk of all present pulmonary veins were dissected together with their direct tributaries. Dissected veins were cut off from the rest of the pulmonary vasculature by a single incisions. After dissection, all hearts were fixed in 10% paraformaldehyde solution minimum for a one month and maximum of two months, until the measurements were performed. The hearts were weighted and the heart circumference (the smallest circumference of the heart measured between both atria and ventricles in the place of the coronary sulcus) was obtained before fixation.

All 130 heart specimens were opened in the usual routine manner with an incision along the posterior wall of the LA, perpendicularly to the mitral valve, half-way between left and right PVs ostia.

All measurements were conducted using a 0.03 mm precision electronic YATO caliper (YT–7201). All measurements were performed by two researchers in order to reduce chances of human error. If the measurements of one parameter differed by more than 10%, they were not included in the database and the sample was measured again until the full compatibility between researchers. The mean of the two measurements was calculated, with approximation to the tenths decimal place.

Number, locations, variations and morphology of PV ostia were recorded in every heart. The transverse diameter of all PV ostia, which is the largest dimension up to the first point of resistance, was measured. The diameter was measured in one plane (the same in every ostium), parallel to the line determined by the coronary sinus. The ostium-to-last-tributary distance was measured as the shortest distance from the PV ostium to the distal junction of its last tributary (the nearest to the ostium), along the course of the PV. The macroscopic presence of myocardial sleeves was evaluated.

### Statistical analysis

StatSoft Statistica 10.0 for Windows was used for all statistical analyses. The data are presented as mean values, with corresponding standard deviations and percentages. The independent *t*-test, paired-samples *t*-test, the Mann–Whitney *U*-test, Wilcoxon signed rank test were executed for the statistical comparison of PV ostium diameter and ostium-to-last-tributary distance between hearts. Correlation coefficients were calculated to measure statistical dependence. *P*-values lower than 0.05 were considered as statistically significant.

## Results

Mean heart weight was 448.6 ± 121.1 g and mean heart circumference was 24.1 ± 2.9 cm. Fife-hundred-forty-one PV ostia in 130 hearts were identified and measured.

### Four classical PVs pattern

**Figure 1:**
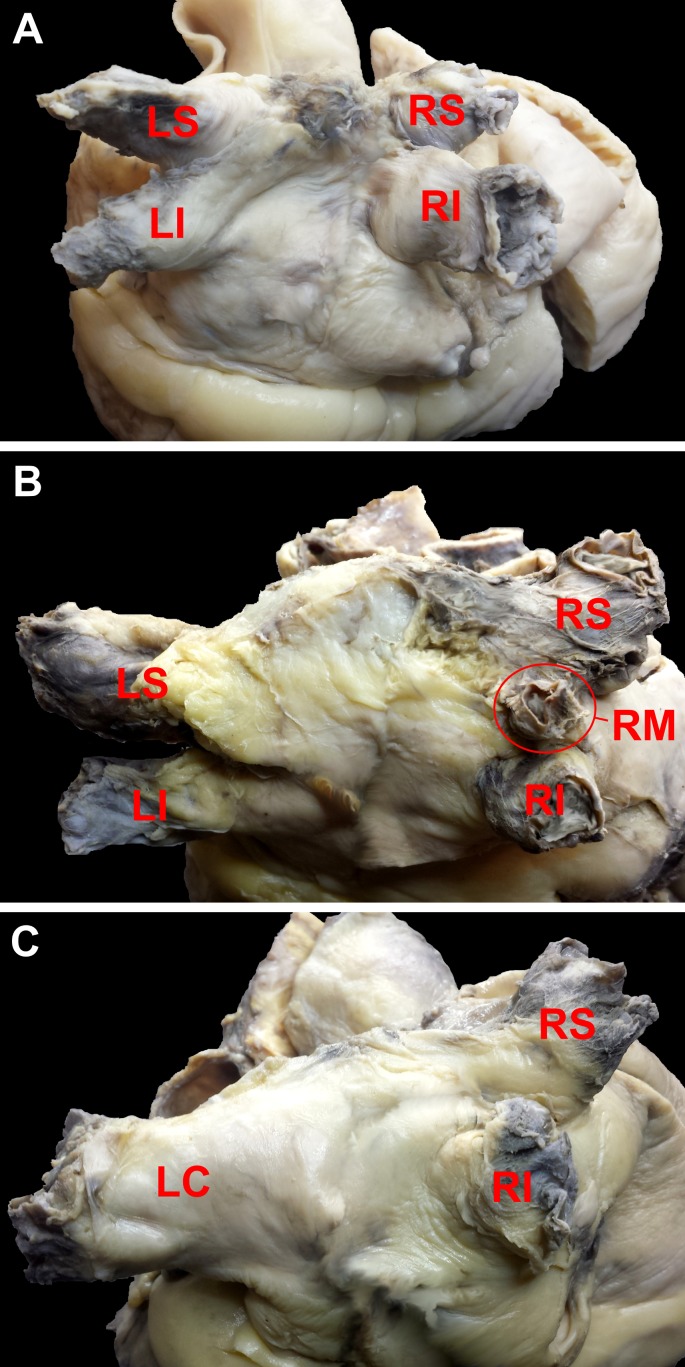
Photograph of cadaveric heart specimens with examples of pulmonary veins ostia patterns (view of the posterior and superior wall of the left atrium). (A) four classical pulmonary veins pattern; (B) additional middle right pulmonary vein; (C) common ostium for left superior and inferior pulmonary vein. LC, left common; LI, left inferior; LS, left superior; RI, right inferior; RM, right middle; RS, right superior.

Four classical PV ostia pattern (two left and two right PVs) were observed in 92 (70.8%) cases. Table 1 presents mean, minimum and maximum values of PV ostia diameters in hearts with 4 classical PVs pattern. There were no significant differences in PV diameter between sexes. Mean diameters for the right superior and the right inferior PVs were correlated with age (*r* = 0.22; *p* = 0.03 and *r* = 0.29; *p* = 0.005 respectively). The diameters were not dependent on BMI, heart weight and heart circumference. The ostium diameters are significantly larger for the superior PVs than the inferior PVs (*p* < 0.05). In 29.2% of hearts, PVs anatomy was different than typically. In 19.2% of our samples additional PV was located on the right and on the left side only in 4.6% (Table 2). Figure 1 presents the most common variations.

**Table 1:** Mean, minimum and maximum values of pulmonary veins ostia diameters (mm).

Group	*N*	Pulmonary vein	Ostium diameter (mm)			
			Mean	SD	Minimum	Maximum
Classical 4 PVs pattern	92	Left superior	13.8	2.9	6.2	19.8
		Left inferior	13.3	3.4	4.4	27.1
		Right superior	14.3	2.9	7.6	21.7
		Right inferior	13.7	3.3	5.3	22.5
Additional middle right PV	25	Left superior	13.2	2.9	8.3	21.2
		Left inferior	12.0	3.1	3.7	19.6
		Right superior	12.7	3.7	6.4	20.0
		Right inferior	12.4	3.9	4.9	19.9
		Additional right middle	8.2	4.1	2.1	22.0
Common ostium for left superior and inferior PVs	6	Common left	19.6	6.7	10.3	26.3
		Right superior	13.1	5.1	7.2	20.2
		Right inferior	13.7	2.1	12.0	17.4

**Notes.**

N Number of samples PV Pulmonary vein SD Standard deviation

**Table 2:** All pulmonary vein ostium variations observed in this study.

Group	*N*	%
Four classical PVs pattern	92	70.8%
Additional middle right PV	25	19.2%
Common ostium for the left superior and left inferior PVs	6	4.44%
Common ostium for the left superior and left the inferior PVs, with additional middle right PV	2	1.48%
Lack of the left inferior PV	1	0.77%
Two additional middle veins (one left and one right)	1	0.77%
Four right PVs with two left PVs	1	0.77%
Lack of the right and left inferior PVs	1	0.77%
Additional middle left PV	1	0.77%

**Notes.**

N Number of samples PV Pulmonary vein

### Heart with 2 PV ostia

There was only one heart (0.77%) with two ostia. In this case, lack of inferior PVs ostia—both left and right ones—was noticed. The diameters of the right and the left PVs ostia were 12.0 mm and 10.7 mm respectively.

### Hearts with 3 PV ostia

This group contains 7 heart samples (5.38%) with two different variations of the PV ostia pattern:

I—common ostium for the left superior and the left inferior PV (4.44%),

II—lack of the left inferior PV ostium (0.77%).

The mean diameter of the common ostium for the left superior and the left inferior PV was 19.6 ± 6.7 mm and was significantly higher than mean diameters for the left superior, the right superior and the right inferior PVs ostia in classical pattern (*p* < 0.05)— Table 1.

### Hearts with 4 PV ostia (with variations)

In two cases (1.48%) we observed 4 ostia of the PVs, but the anatomy was surprisingly complex. There were two abnormalities found in both hearts: the common ostium for the left superior and left inferior PVs, as well as the additional middle right PV ostium.

### Hearts with 5 PV ostia

There were 26 (20% of total amount) cases in which an extra PV ostium was described. In 25 of them an additional middle right PV ostium was observed (19.2% of all cases). Mean, minimum and maximum values of PV ostia diameters for hearts with additional middle right PV are presented in Table 1. In one heart (0.77%), an additional PV ostium was located on the left side. There were significant differences in diameters of the right superior PV ostium for hearts with an additional middle right PV and hearts with the classical PV pattern (*p* < 0.05); the right superior PV ostium diameter was smaller in hearts with 5 ostia. The mean diameter of the additional middle right PV ostium was the smallest PV ostium diameter in the heart when the additional ostium was present. No significant differences were noticed in PV ostium diameter between sexes. Diameters were not dependent on age, BMI, heart weight nor heart circumference.

### Hearts with 6 PV ostia

Only 2 hearts (1.54%) had two additional PVs ostia. In one of them, we noticed four right PVs, with two regular left ones. In the latter case, two additional middle veins were observed: one left and one right.

### Ostium-to-last-tributary distance

**Figure 2:**
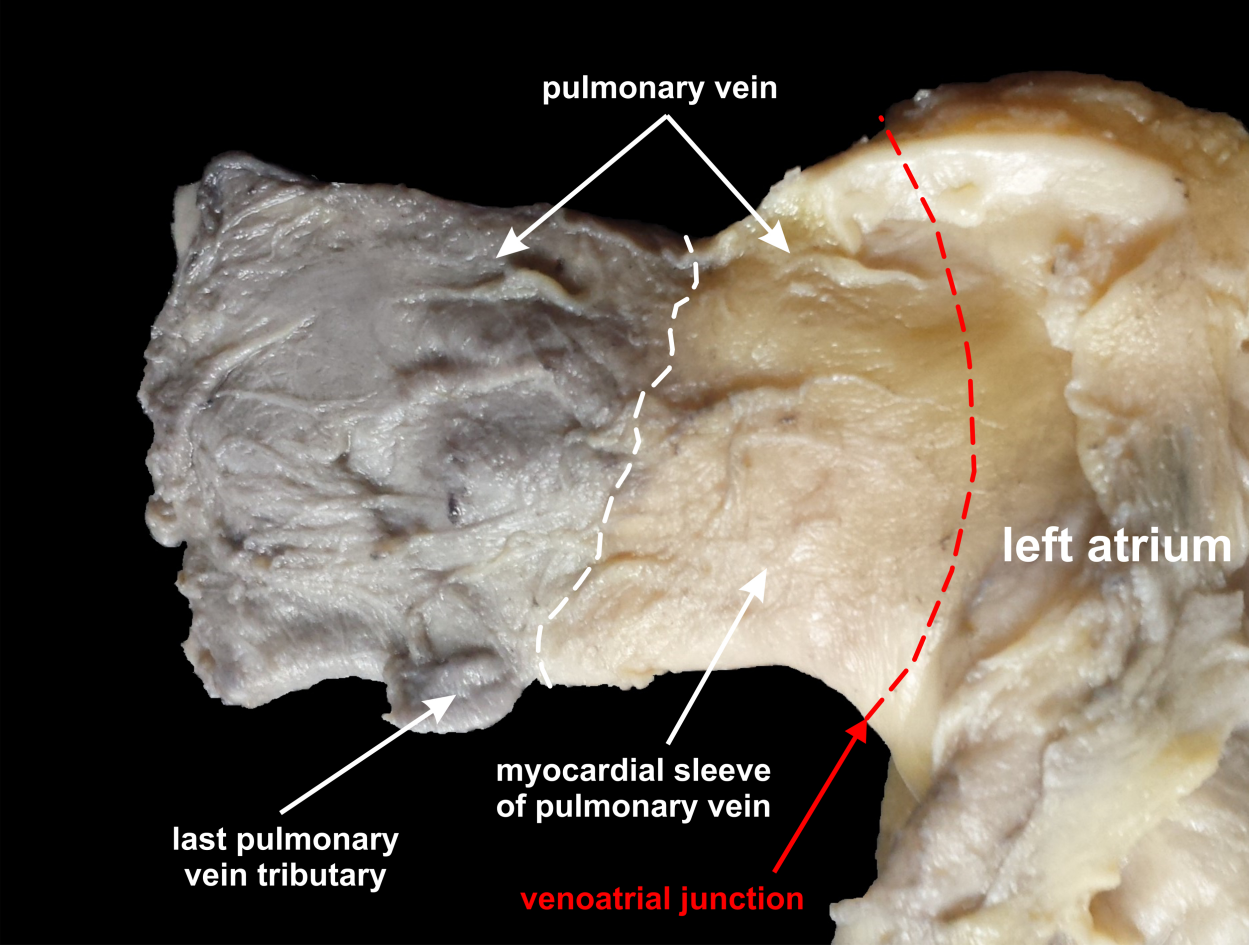
Photograph of cadaveric heart specimen. Left superior pulmonary vein with clearly expressed myocardial sleeves of pulmonary vein.

Table 3 shows mean, maximum and minimum values of the ostium-to-last-tributary distance. The ostium-to-last-tributary distance of the left superior and the left inferior PVs (for classical pattern) were correlated with age (*r* = 0.31; *p* = 0.003 and *r* = 0.23; *p* = 0.03 respectively) and BMI (*r* = 0.24; *p* = 0.02 and *r* = 0.25; *p* = 0.02 respectively). The distance was not dependent on heart weight, heart circumference and PV ostium diameter for classical PV ostium pattern and also not dependent on age, BMI, heart weight, heart circumference and PV ostium diameter for hearts with additional middle right PV. There were no significant differences in ostium-to-last-tributary distance between sexes. In all 130 investigated hearts, we were able to detect myocardial sleeves macroscopically which were extended from the LA onto the adventitial surface of the PVs. They could be detected due to difference in color and course of fibers (Fig. 2).

**Table 3:** Mean, minimum and maximum values of the ostium-to-last-tributary distance (mm).

Group	*N*	Pulmonary vein	Ostium-to-last-tributary distance (mm)			
			Mean	SD	Minimum	Maximum
Classical 4 PVs pattern	92	Left superior	15.1	4.6	8.0	27.0
		Left inferior	13.5	4.0	6.1	22.3
		Right superior	11.8	4.0	3.8	22.0
		Right inferior	11.0	3.7	4.0	22.2
Additional middle right PV	25	Left superior	14.6	5.7	7.9	31.3
		Left inferior	14.2	7.1	5.9	37.7
		Right superior	11.7	3.0	7.5	19.0
		Right inferior	11.4	4.0	6.4	23.0
		Additional right middle	7.8	3.2	4.0	17.7
Common ostium for left superior and inferior PVs	6	Common left	16.9	6.9	6.6	27.0
		Right superior	10.5	2.5	7.3	14.1
		Right inferior	10.7	2.2	8.9	14.4

**Notes.**

*N* Number of samples PV Pulmonary vein SD Standard deviation

## Discussion

There is no doubt that the clinical importance of pulmonary vein anatomy is crucial for planning and performing the catheter and balloon-based pulmonary vein ablation (isolation) procedures (Aktan Ikiz, Üçerler & Özgür, 2014; Köse et al., 2012; Mayor, 2015; Nalliah et al., 2015; Rettmann et al., 2015; Sarabanda et al., 2005; Stanford & Breen, 2005). Classical 4 PV ostia pattern could be observed in 68–81% of all individuals (Aktan Ikiz, Üçerler & Özgür, 2014; Kaseno et al., 2008; L C et al., 2014; Marom et al., 2004; Scharf et al., 2003; Tekbas et al., 2012). The present study confirms that a common PV ostium occurring on the left side is the most frequent anatomical variation in the healthy population, whereas an additional ostium is most common on the right side of the left atrium (Prasanna et al., 2014; Stanford & Breen, 2005; Vasamreddy et al., 2004; Woźniak-Skowerska et al., 2011). Contrast-enhanced computed tomography, PV angiography, magnetic resonance imaging, transthoracic, transoesophageal or intra-cardiac echocardiography are extremely helpful, commonly used techniques before radiofrequency catheter ablation/isolation of PVs. Diagnostic imaging performed prior catheter procedures to provide accurate elucidation of the anatomy is the only way for their safe and effective execution (Stanford & Breen, 2005; Vasamreddy et al., 2004).

Our study confirms that the accessory middle right PV is the second most common variation immediately after the classical pattern in healthy individuals (Ho, Anderson & Sánchez-Quintana, 2002; Niculescu et al., 2006; Stanford & Breen, 2005). This additional vein (always with the smallest diameter among all PVs) is the middle lobe vein which drains directly to the left atrium. Originally, the middle lobe vein is tributary to the superior pulmonary vein, but in about 20% of all patients it can pass independently. Since this vein also has the myocardial sleeve, clinicians should be aware of this frequent anomaly, especially since its minimum diameter is only about 2 mm, and it could be omitted in imaging tests.

Some studies pointed that there is no significant association between any particular PV drainage pattern and atrial arrhythmia. However, Marom et al. (2004) stated that patients with the additional right middle lobe PV tended to present higher frequency of AF than those with other patterns. Other studies show that anatomical variants are observed more often in patients with AF compared to controls (Gebhard et al., 2014). Den Uijl et al. (2011) stated that the presence of normal anatomy of the right PVs is an independent risk factor for AF recurrence. Interestingly, we can find some imaging studies which indicates that the classical PV ostium pattern could be seen in the minority of patients with AF undergoing PV isolation (McLellan et al., 2014; Mlcochová et al., 2005). Mlcochová et al. (2005) have shown the classical pattern in only 30% and the presence of a left common PV ostium in 75% of patients using 3D magnetic resonance angiography. Also, McLellan et al. noticed the classical pattern and the left common PV ostium in 47% and 37% of AF patients respectively; they also concluded that the presence of the left common PV was associated with an increased freedom from AF after catheter ablation (assessed by using Cox regression analysis in univariate and multivariate models) (McLellan et al., 2014). In conclusion, we can note a trend that the common left PV ostia are more frequently found in AF patients, while in the normal population they are the minor variation (up to 15%) (Aktan Ikiz, Üçerler & Özgür, 2014; Woźniak-Skowerska et al., 2011). Isolation of such small ostia entails much higher risk of PV stenosis caused by catheter ablation.

In our study group, we noted that the ostium diameters are significantly larger for the superior PVs than the inferior PVs. There were no relations found between the PV dimensions and outcome of PVs radiofrequency catheter ablation in previous studies (Den Uijl et al., 2011; Hof et al., 2009). Woźniak-Skowerska et al. (2011) found that mean diameters of all PVs (except for the right inferior PV) were larger in AF patients than in control cases. Gebhard et al. (2014) and Schwartzman, Lacomis & Wigginton (2003) confirmed increased diameter of PV ostia in the AF group and also the total ostial PV volume is significantly increased in patients with AF, compared to controls. Mean PV diameters at the ostia were slightly higher in our study group, compared with the studies conducted by Cronin et al. (2009) and Kim et al. (2005), and lower relative to Schwartzman, Lacomis & Wigginton (2003) and Gebhard et al. (2014). What is surprising is that PV diameters measured by Niculescu et al. (2006) were over 2.5 times higher than our results. There is also evidence that ostium diameters are much larger in male PVs than in the female ones, but we cannot confirm those statements (Schwartzman, Lacomis & Wigginton, 2003). Kato et al. (2003) draws the attention to the shape of the PV ostia. In both AF patients and control group, ostia were oblong in shape with the superior-inferior dimension greater than the anteroposterior dimension (Kato et al. 2003). We can confirm that PV ostia are not perfectly round, but more oval in shape.

Ostium-to-last-tributary distance (also known in other studies as the distance to the first bifurcation or branch) could be a very informative parameter for clinicians. This study shows that myocardial sleeves of PVs are macroscopically present in all pulmonary veins. Our mean measurement results of ostium-to-last-tributary distance were significantly lower in comparison with the computed tomography study conducted by Cronin et al. (2009) (except for the right inferior PV, where it was significantly higher) and 2–3 times lower when compared with Schwartzman, Lacomis & Wigginton (2003) for both AF (−) and AF (+) groups (multidimensional computed tomography).

There are some limitations of this study. The first is that our study comprised only cases with no history of AF, but it can also be considered as an advantage, since we are able to show anatomical variations in healthy group which may be used as a control for other studies. The second is that all the measurements were taken from autopsied heart specimens that were fixed in formalin, which could cause some slight changes in size and shape of the heart.

## Conclusion

Only 71% of the investigated hearts contained four standard pulmonary veins. The middle right pulmonary vein is present in almost 20% of the hearts. Presented data can provide useful information for the clinicians during interventional procedures or radiologic examinations of PVs.

## Supplemental Information

10.7717/peerj.1579/supp-1 Supplemental Information 1 Dataset—pulmonary veins anatomy

## Additional Information and Declarations

### Data Availability

The following information was supplied regarding data availability: Data is available in the Supplemental Information.

## References

[ref-1] Aktan Ikiz ZA, Üçerler H, Özgür T (2014). Anatomic characteristics of left atrium and openings of pulmonary veins. Anadolu Kardiyoloji Dergisi.

[ref-2] Chen SA, Tai CT, Tsai CF, Hsieh MH, Ding YA, Chang MS (2000). Radiofrequency catheter ablation of atrial fibrillation initiated by pulmonary vein ectopic beats. Journal of Cardiovascular Electrophysiology.

[ref-3] Cronin P, Saab A, Kelly AM, Gross BH, Patel S, Kazerooni EA, Carlos RC (2009). Measurements of pulmonary vein ostial diameter and distance to first bifurcation: a comparison of different measurement methods. European Journal of Radiology.

[ref-4] Den Uijl DW, Tops LF, Delgado V, Schuijf JD, Kroft LJ, de Roos A, Boersma E, Trines SA, Zeppenfeld K, Schalij MJ, Bax JJ (2011). Effect of pulmonary vein anatomy and left atrial dimensions on outcome of circumferential radiofrequency catheter ablation for atrial fibrillation. American Journal of Cardiology.

[ref-5] Fynn SP, Kalman JM (2004). Pulmonary veins: anatomy, electrophysiology, tachycardia, and fibrillation. Pacing and Clinical Electrophysiology.

[ref-6] Gebhard C, Krasniqi N, Stähli BE, Klaeser B, Fuchs TA, Ghadri JR, Haegeli L, Lüscher TF, Kaufmann PA, Duru F (2014). Characterization of pulmonary vein dimensions using High-definition 64-Slice computed tomography prior to radiofrequency catheter ablation for atrial fibrillation. Cardiology Research and Practice.

[ref-7] Gill JS (2004). How to perform pulmonary vein isolation. Europace.

[ref-8] Haïssaguerre M, Jaïs P, Shah DC, Takahashi A, Hocini M, Quiniou G, Garrigue S, Le Mouroux A, Le Métayer P, Clémenty J (1998). Spontaneous initiation of atrial fibrillation by ectopic beats originating in the pulmonary veins. New England Journal of Medicine.

[ref-9] Ho SY, Anderson RH, Sánchez-Quintana D (2002). Atrial structure and fibres: morphologic bases of atrial conduction. Cardiovascular Research.

[ref-10] Hof I, Chilukuri K, Arbab-Zadeh A, Scherr D, Dalal D, Nazarian S, Henrikson C, Spragg D, Berger R, Marine J, Calkins H (2009). Does left atrial volume and pulmonary venous anatomy predict the outcome of catheter ablation of atrial fibrillation?. Journal of Cardiovascular Electrophysiology.

[ref-11] Jaïs P, Haïssaguerre M, Shah DC, Chouairi S, Gencel L, Hocini M, Clémenty J (1997). A focal source of atrial fibrillation treated by discrete radiofrequency ablation. Circulation.

[ref-12] Kaseno K, Tada H, Koyama K, Jingu M, Hiramatsu S, Yokokawa M, Goto K, Naito S, Oshima S, Taniguchi K (2008). Prevalence and characterization of pulmonary vein variants in patients with atrial fibrillation determined using 3-dimensional computed tomography. American Journal of Cardiology.

[ref-13] Kato R, Lickfett L, Meininger G, Dickfeld T, Wu R, Juang G, Angkeow P, LaCorte J, Bluemke D, Berger R, Halperin HR, Calkins H (2003). Pulmonary vein anatomy in patients undergoing catheter ablation of atrial fibrillation: lessons learned by use of magnetic resonance imaging. Circulation.

[ref-14] Kim YH, Marom EM, Herndon JE, McAdams HP (2005). Pulmonary vein diameter, cross-sectional area, and shape: CT analysis. Radiology.

[ref-15] Köse S, Başarıcı I, Kabul KH, Bozlar U, Amasyalı B (2012). Catheter ablation of atrial fibrillation in a patient with unusual pulmonary vein anatomy involving right upper pulmonary vein. Anadolu Kardiyol Derg.

[ref-16] L C P, R P, D’Souza AS, Bhat KM (2014). Variations in the pulmonary venous ostium in the left atrium and its clinical importance. Journal of Clinical and Diagnostic Research.

[ref-17] Marom EM, Herndon JE, Kim YH, McAdams HP (2004). Variations in pulmonary venous drainage to the left atrium: implications for radiofrequency ablation. Radiology.

[ref-18] Mayor S (2015). Ablation of pulmonary veins works as well as more extensive treatment in persistent atrial fibrillation, study finds. BMJ.

[ref-19] McLellan AJ, Ling LH, Ruggiero D, Wong MC, Walters TE, Nisbet A, Shetty AK, Azzopardi S, Taylor AJ, Morton JB, Kalman JM, Kistler PM (2014). Pulmonary vein isolation: the impact of pulmonary venous anatomy on long-term outcome of catheter ablation for paroxysmal atrial fibrillation. Heart Rhythm.

[ref-20] Mlcochová H, Tintera J, Porod V, Peichl P, Cihák R, Kautzner J (2005). Magnetic resonance angiography of pulmonary veins: implications for catheter ablation of atrial fibrillation. Pacing and Clinical Electrophysiology.

[ref-21] Moubarak JB, Rozwadowski JV, Strzalka CT, Buck WR, Tan WS, Kish GF, Kisiel T, Fronc HC, Maloney JD (2000). Pulmonary veins-left atrial junction: anatomic and histological study. Pacing and Clinical Electrophysiology.

[ref-22] Nalliah CJ, Lim TW, Kizana E, Qian P, Kovoor P, Thiagalingam A, Ross DL, Thomas SP (2015). Clinical significance of early atrial arrhythmia type and timing after single ring isolation of the pulmonary veins. Europace.

[ref-23] Niculescu MC, Niculescu V, Sişu AM, Ciobanu IC, Dăescu E, Petrescu CI, Jianu A, Rusu MC (2006). Study of the diameter and number of the pulmonary veins orifices. Romanian Journal of Morphology and Embryology.

[ref-24] Prasanna L, Praveena R, D’Souza A, Bhat K (2014). Variations in the pulmonary venous ostium in the left atrium and its clinical importance. Journal of Clinical and Diagnostic Research.

[ref-25] Rettmann ME, Holmes DR, Breen JF, Ge X, Karwoski RA, Monahan KH, Bahnson TD, Packer DL, Robb RA, Investigators CPI (2015). Measurements of the left atrium and pulmonary veins for analysis of reverse structural remodeling following cardiac ablation therapy. Computer Methods and Programs in Biomedicine.

[ref-26] Saito T, Waki K, Becker AE (2000). Left atrial myocardial extension onto pulmonary veins in humans: anatomic observations relevant for atrial arrhythmias. Journal of Cardiovascular Electrophysiology.

[ref-27] Sarabanda AV, Bunch TJ, Johnson SB, Mahapatra S, Milton MA, Leite LR, Bruce GK, Packer DL (2005). Efficacy and safety of circumferential pulmonary vein isolation using a novel cryothermal balloon ablation system. Journal of the American College of Cardiology.

[ref-28] Scharf C, Sneider M, Case I, Chugh A, Lai SW, Pelosi F, Knight BP, Kazerooni E, Morady F, Oral H (2003). Anatomy of the pulmonary veins in patients with atrial fibrillation and effects of segmental ostial ablation analyzed by computed tomography. Journal of Cardiovascular Electrophysiology.

[ref-29] Schwartzman D, Lacomis J, Wigginton WG (2003). Characterization of left atrium and distal pulmonary vein morphology using multidimensional computed tomography. Journal of the American College of Cardiology.

[ref-30] Stanford W, Breen JF (2005). CT evaluation of left atrial pulmonary venous anatomy. The International Journal of Cardiovascular Imaging.

[ref-31] Tekbas G, Gumus H, Onder H, Ekici F, Hamidi C, Tekbas E, Gulicetincakmak M, Yavuz C, Bilici A (2012). Evaluation of pulmonary vein variations and anomalies with 64 slice multi detector computed tomography. Wien Klin Wochenschr.

[ref-32] Vasamreddy CR, Jayam V, Lickfett L, Nasir K, Bradley DJ, Eldadah Z, Dickfeld T, Donahue K, Halperin HS, Berger R, Calkins H (2004). Technique and results of pulmonary vein angiography in patients undergoing catheter ablation of atrial fibrillation. Journal of Cardiovascular Electrophysiology.

[ref-33] Woźniak-Skowerska I, Skowerski M, Wnuk-Wojnar A, Hoffmann A, Nowak S, Gola A, Sosnowski M, Trusz-Gluza M (2011). Comparison of pulmonary veins anatomy in patients with and without atrial fibrillation: analysis by multislice tomography. International Journal of Cardiology.

